# Biocompatibility and colorectal anti-cancer activity study of nanosized BaTiO_3_ coated spinel ferrites

**DOI:** 10.1038/s41598-022-18306-5

**Published:** 2022-08-19

**Authors:** Tahani M. Alfareed, Yassine Slimani, Munirah A. Almessiere, Muhammad Nawaz, Firdos A. Khan, Abdulhadi Baykal, Ebtesam A. Al-Suhaimi

**Affiliations:** 1 Master Program of Nanotechnology, Institute for Research and Medical Consultations (IRMC), Imam Abdulrahman Bin Faisal University, P.O. Box 1982, Dammam, 31441 Saudi Arabia; 2 Department of Biophysics, Institute for Research and Medical Consultations (IRMC), Imam Abdulrahman Bin Faisal University, P.O. Box 1982, Dammam, 31441 Saudi Arabia; 3 Department of Physics, College of Science, Imam Abdulrahman Bin Faisal University, P.O. Box 1982, Dammam, 31441 Saudi Arabia; 4 Department of Nanomedicine Research, Institute for Research and Medical Consultations (IRMC), Imam Abdulrahman Bin Faisal University, P.O. Box 1982, Dammam, 31441 Saudi Arabia; 5 Department of Stem Cells, Institute for Research and Medical Consultations (IRMC), Imam Abdulrahman Bin Faisal University, P.O. Box 1982, Dammam, 31441 Saudi Arabia; 6 Biology Department, College of Science & Institute for Research and Medical Consultations (IRMC), Imam Abdulrahman Bin Faisal University, P.O. Box 1982, Dammam, 31441 Saudi Arabia

**Keywords:** Biological techniques, Nanobiotechnology, Nanoscience and technology, Nanomedicine, Nanoscale materials

## Abstract

In the present work, different nanoparticles spinel ferrite series (MFe_2_O_4_, Co_0.5_M_0.5_Fe_2_O_4_; M = Co, Mn, Ni, Mg, Cu, or Zn) have been obtained via sonochemical approach. Then, sol–gel method was employed to design core–shell magnetoelectric nanocomposites by coating these nanoparticles with BaTiO_3_ (BTO). The structure and morphology of the prepared samples were examined by X-ray powder diffraction (XRD), scanning electron microscope (SEM) coupled with energy dispersive X-ray spectroscopy (EDX), high-resolution transmission electron microscope (HR-TEM), and zeta potential. XRD analysis showed the presence of spinel ferrite and BTO phases without any trace of a secondary phase. Both phases crystallized in the cubic structure. SEM micrographs illustrated an agglomeration of spherical grains with nonuniformly diphase orientation and different degrees of agglomeration. Moreover, HR-TEM revealed interplanar d-spacing planes that are in good agreement with those of the spinel ferrite phase and BTO phase. These techniques along with EDX analyses confirmed the successful formation of the desired nanocomposites. Zeta potential was also investigated. The biological influence of (MFe_2_O_4_, CoMFe) MNPs and core–shell (MFe_2_O_4_@BTO, CoMFe@BTO) magnetoelectric nanocomposites were examined by MTT and DAPI assays. Post 48 h of treatments, the anticancer activity of MNPs and MENCs was investigated on human colorectal carcinoma cells (HCT-116) against the cytocompatibility of normal non-cancerous cells (HEK-293). It was established that MNPs possess anti-colon cancer capability while MENCs exhibited a recovery effect due to the presence of a protective biocompatible BTO layer. RBCs hemolytic effect of NPs has ranged from non- to low-hemolytic effect. This effect that could be attributed to the surface charge from zeta potential, also the CoMnFe possesses the stable and lowest zeta potential in comparison with CoFe_2_O_4_ and MnFe_2_O_4_ also to the protective effect of shell. These findings open up wide prospects for biomedical applications of MNPs as anticancer and MENCs as promising drug nanocarriers.

## Introduction

Nanoparticles are well known as drug delivery systems in biomedicine as they can conquer biological barriers, minimize doses of the drug that must be given^1^ and reduce side effects. Magnetoelectric nanocomposites (MENCs) are the latest development in the technology of magnetic nanoparticles. MENCs possess both properties of magnetic and novel electric properties^2^. The mechanism of action of MENCs in the biological environment mainly relays on the formation of the pores on cancer cells^3^. The electrical properties Vm of cancer cells differ from their counterparts’ healthy cells. Tumor cells exhibited distinctive bioelectrical characteristics where electrophysiological analysis of different tumor cells showed a depolarization (i.e. less negative) that favors and as a property of a fast cellular growing state^4–6^. The depolarized membrane potential makes tumor cells more susceptible to electroporation, permitting the delivery inside the cells through the produced pores^7^. The generated electric field by MENCs can be variated through many parameters one of them is the type of magnetic phase (core) in core–shell MENCs.

Barium titanate, BaTiO_3_ (noted BTO), is a smart material that exhibits a piezoelectric characteristic through the generation of electrical polarization in response to minute structural deformations^8^. It has been stated that BTO possesses biological characteristics including high biocompatibility when contacted with biological cells. Therefore, it has been considered as a promising material in biomedicine applications^9^. Ciofani et al. have reported the cytocompatibility of BTO NPs at higher concentrations such as 100 μg/ml on mesenchymal stem cells (MSCs)^10^. According to Ref.^11^, poly(lactic-*co*-glycolic) acid/BTO NPs have shown their role in cell attachment and the effects on the differentiation and proliferation of osteoblast and osteocytes.

Spinel ferrite is the most attractive group of iron oxide materials due to the diversity in the chemical composition leading to a broad range of physical characteristics in a variety of applications^12–15^. The structure of spinel ferrite consists of a cubic close-packed arrangement of oxygen ions with total 56 atoms that are subdivided into 32 O^2−^ anions and 24 cations. The spinel ferrite structure possesses two crystallographic sites where 8 A-sites are occupied by tetrahedrally coordinated cations and 16 B-sites are octahedrally coordinated^16^. The spinel magnetic properties are governed by the type of metal cations and their distribution between the two crystallographic sites^17,18^. The metal cations distribution is affected by several factors including the ionic radii of cations, size of the interstitial site, stabilization energy, preparation method, and the reaction conditions^19^. The magnetic materials are divided based on their capability to be magnetized and demagnetized. In general, there are two types of magnetic materials which are hard and soft magnets. Hard magnets retain permeant magnetization in the absence of an applied field, while soft magnets are easy to magnetize and demagnetize.

Magnetic nanoparticles possess a considerable interest in biomedical applications for diagnosis and cancer therapy^20^. Magnetic nanoparticles are capable to act as a drug delivery system^21,22^ where it accumulates at the tumor sites through passive or active targeting. Passive targeting mostly relays on exploiting the enhanced permeability and retention (EPR) effect, due to the leaky nature and physiologically defective tumor vasculature as well as the lack of a lymphatic system for drainage^23^. On contrary, active targeting is based on the magnetic response of nanoparticles via applied magnetic fields. Hyperthermia is another cancer therapy technique where the cancer cells can be destroyed when subjected to high temperatures (40–45 °C)^24–27^. Magnetic nanoparticles produce heat when exposed to an alternating magnetic field due to relaxations of rotating magnetic moment^20^. Moreover, magnetic nanoparticles have been utilized as enhanced contrast agents in magnetic resonance imaging (MRI)^28^.

The potential practical bio-applications of nanoparticles can be considered only when their toxicity is very well understood. In particular, each time a new nanomaterial aimed for biomedical applications required an extensive examination of its biosafety. Hemolysis is a considerable blood compatibility analysis as the nanoparticles could be directly contacted with red blood cells (RBC) via bloodstream injection. Hemolysis occurs when the RBC membrane is damaged, leading to leakage of hemoglobin. This causes several adverse health effects such as renal toxicity, hypertension, and anemia. Furthermore, the other blood compartments [platelets and white blood cells (WBC)] can be also affected through intravascular hemolysis which leads to coagulation, or immune deficiency^29,30^. Several reports have shown that Fe_3_O_4_, ZnFe_2_O_4_, CaFe_2_O_4_, CuFe_2_O_4_, MgFe_2_O_4_, NiFe_2_O_4_, and MnFe_2_O_4_ MNPs exhibited a toxic effect when used above 10 µg/0.1 ml concentration^31–35^, while Ca_x_Mg_x_Ni_1−2x_Fe_2_O_4_ (x ≤ 0.05) NPs have shown a reduction in cell viability at 100 µg/0.1 ml^36^. The nanoparticles-cell interaction can be initiated by adhering the nanoparticles to the cell surface, then are internalized via endocytosis, and amassed inside digestive vacuoles. Thus, it is very likely to happen cytotoxicity at higher concentrations due to particle overload to the cells^32^.

To our knowledge, there is no evidence has been found in the literature on bioactivities examination of core–shell (MFe_2_O_4_@BTO, Co_0.5_M_0.5_Fe_2_O_4_@BTO; where M = Mn, Ni, Mg, Cu, Zn, or Co) MENCs on human colorectal cancer (HCT-116) and human embryonic kidney (HEK-293) cell lines. Thus, this study aims to confirm that MNPs and MENCs do not impact harmful effects on healthy cultured cells and do not promote the growth of cancer cells. We have prepared MNPs and MENCs by sonochemical and sol–gel synthesis approaches, respectively. The surface and structural characterizations were investigated through XRD, SEM, EDX, TEM, and zeta potential procedures. Next, the preliminary in vitro assessment of cytocompatibility and cell viability have been conducted through MTT assay, nuclear DAPI staining, and hemolysis analysis on HCT-116, HEK-293, and RBCs with a special focus on the protective properties of BTO on the used cells.

## Results and discussion

### XRD structural analysis

**Figure 1:**
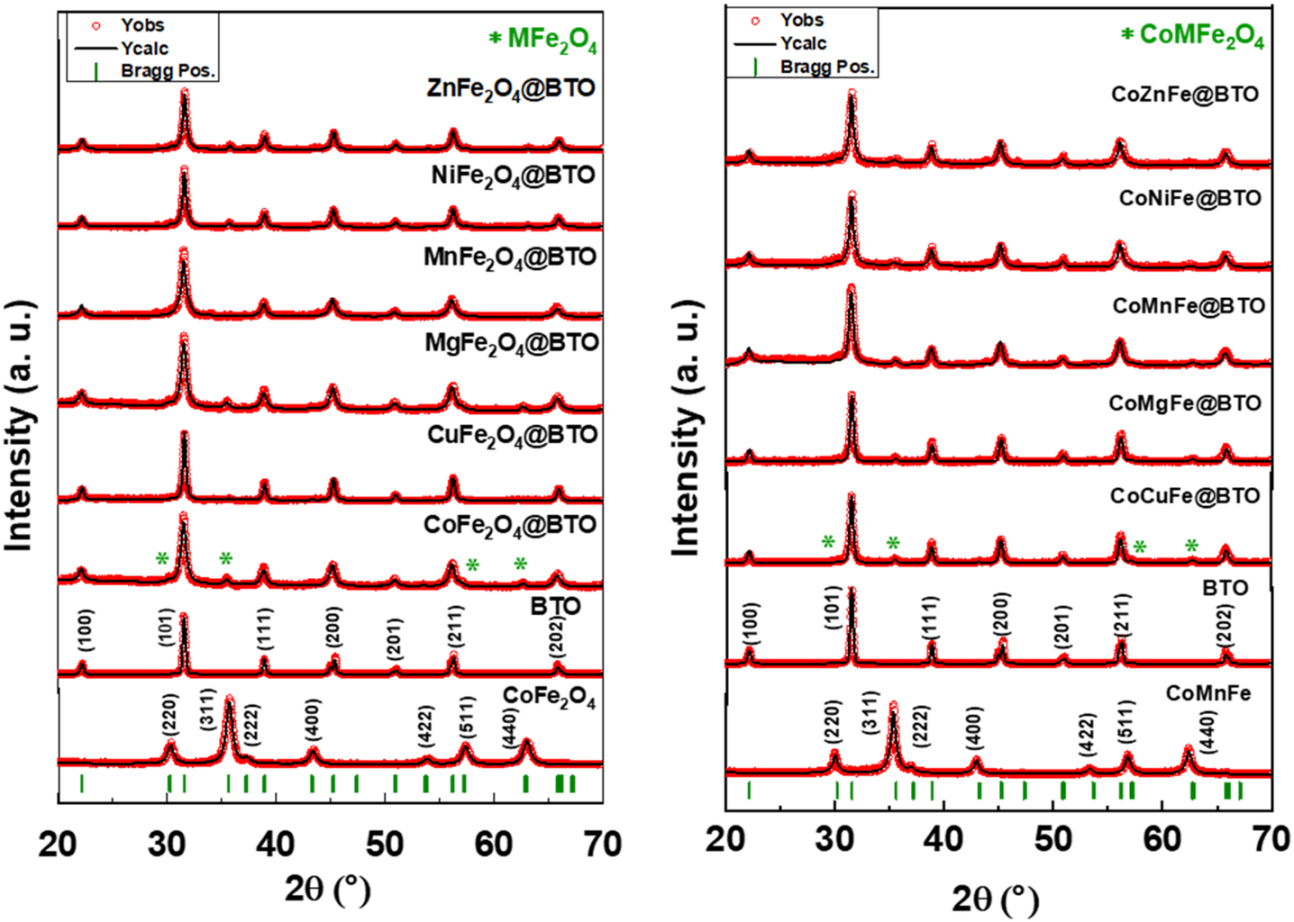
Refined XRD powder patterns of (CoFe_2_O_4_, CoMnFe) MNPs and core–shell (MFe_2_O_4_@BTO, CoMFe@BTO; M = Mn, Ni, Mg, Cu, Zn, or Co) MENCs.

Figure 1 represented the XRD patterns of prepared spinel ferrite (CoFe_2_O_4_, CoMnFe) MNPs and core–shell MENCs (MFe_2_O_4_@BTO, CoMFe@BTO; M = Mn, Ni, Mg, Cu, Zn, or Co). The XRD exhibited the pure spinel ferrite and core–shell structure without any trace of impurity phases. It demonstrated the characteristic peaks of spinel planes for (CoFe_2_O_4_, CoMnFe) which are indexed as (220), (311), (222), (400), (422), (511), and (440). The recorded peaks of the spinel were well-matched with the cubic structure and space group Fd-3m of spinel ferrite according to card No. 96-591-0064^37–40^. Moreover, The XRD of core–shell (MFe_2_O_4_@BTO, CoMFe@BTO, M = Mn, Ni, Mg, Cu, Zn, or Co) MENCs revealed the presence and the combination between two distinct crystallographic orientations (spinel and perovskite phases). The absence of impurities and intermediate phases confirm the successful formation of composite materials as well as the efficiency of the preparation method. The core–shell (MFe_2_O_4_@BTO, CoMFe@BTO) MENCs’ planes are identified as (100), (101), (111), (200), (201), (211), and (202) corresponding to the cubic structure of pure BTO according to the card No.96-210-0863 while the remaining planes (220), (311), (511), and (440) are for (MFe_2_O_4_, CoMFe) MNPs. Herein, the XRD phase identification showed BTO phase matched with the cubic perovskite structure. This was proved by the absence of splitting (200) and (002) peaks and the existence of a single peak at ⁓ 45^41,42^. For a detailed analysis of the structure, Rietveld refinement was performed using a two-phase model of both spinel and BTO phases comparing the experimental diffraction patterns with the standard database through Match3! and Fullproof software to extract the lattice parameter a, unit cell volume V, and crystallite size as listed in Table 1. The average crystallite size (*D*_*XRD*_) of all core–shell MENCs was calculated by considering the most intense peaks (311) and (101) using the famous Debye–Scherrer’s equation and *D*_*XRD*_ values were found in the range of 27–46 nm.

**Table 1:** The lattice parameters and crystallite size of core–shell (MFe_2_O_4_@BTO, CoMFe@BTO; M = Mn, Ni, Mg, Cu, Zn, or Co) MENCs.

Sample	BTO phase		Spinel ferrite phase		D_XRD_ (nm)
	a (Å)	*V* (Å^3^*)*	a (Å)	*V* (Å^3^*)*	
CoFe_**2**_O_4_@BTO	4.0116	64.5579	8.3644	585.2001	45.89
CuFe_**2**_O_4_@BTO	4.0076	64.3655	8.3631	584.9357	43.29
MgFe_**2**_O_4_@BTO	4.0148	64.7130	8.3913	590.8559	27.89
MnFe_**2**_O_4_@BTO	4.0160	64.7701	8.5824	632.1545	27.67
NiFe_**2**_O_4_@BTO	4.0087	64.4175	8.3358	579.2281	37.15
ZnFe_**2**_O_4_@BTO	4.0129	64.6212	8.4372	600.6070	36.78
**Co**_**0.5**_**M**_**0.5**_**Fe**_***2***_**O**_**4**_**@BTO (M = Cu, Mg, Mn, Ni, Zn)**					
CoCuFe@BTO	4.0125	64.6033	8.3721	586.8177	39.70
CoMgFe@BTO	4.0107	64.5174	8.3779	588.0424	40.10
CoMnFe@BTO	4.0177	64.8539	8.3767	587.7772	27.19
CoNiFe@BTO	4.0149	64.7179	8.3448	581.1042	28.54
CoZnFe@BTO	4.0148	64.7145	8.4033	593.4091	30.47

### Morphological and microstructural study

**Figure 2:**
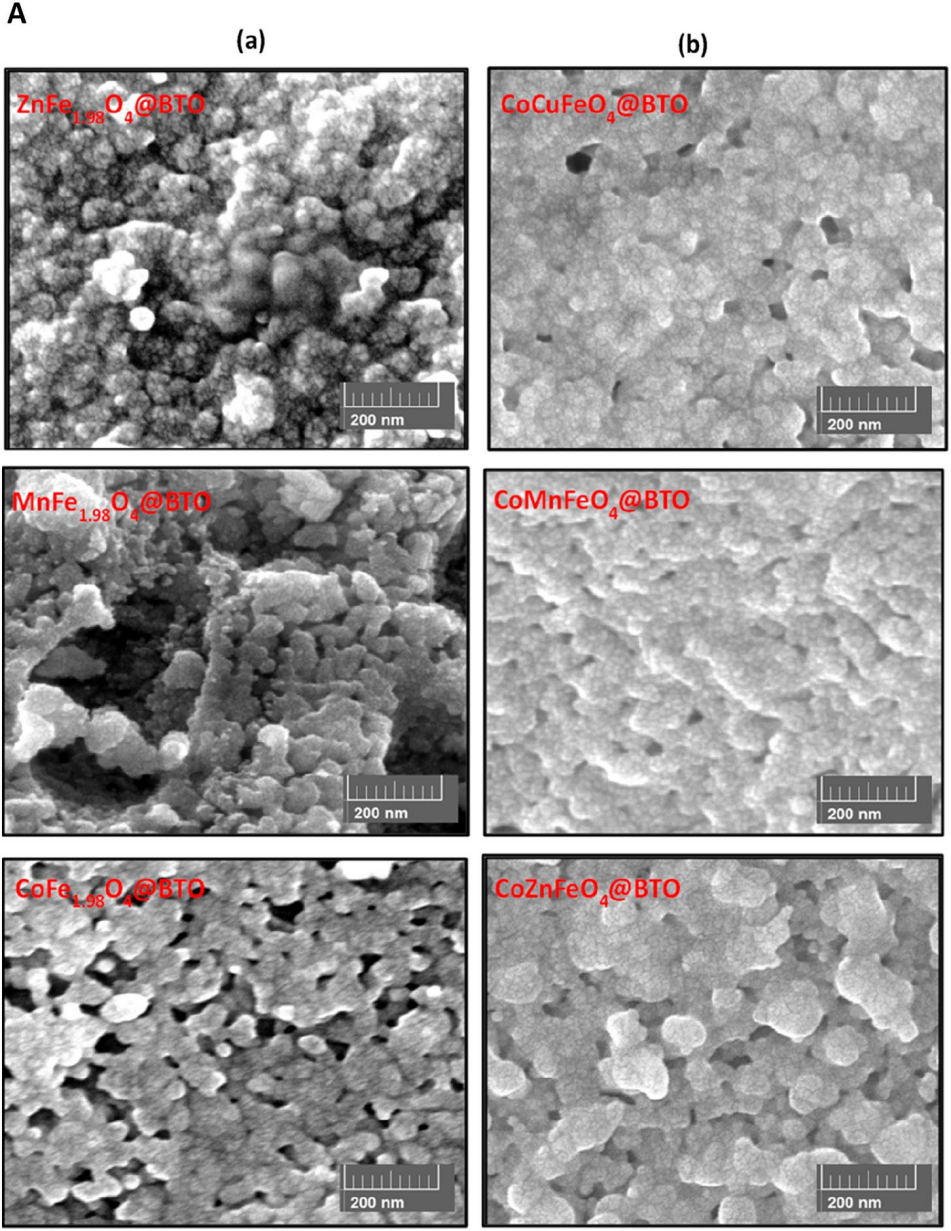

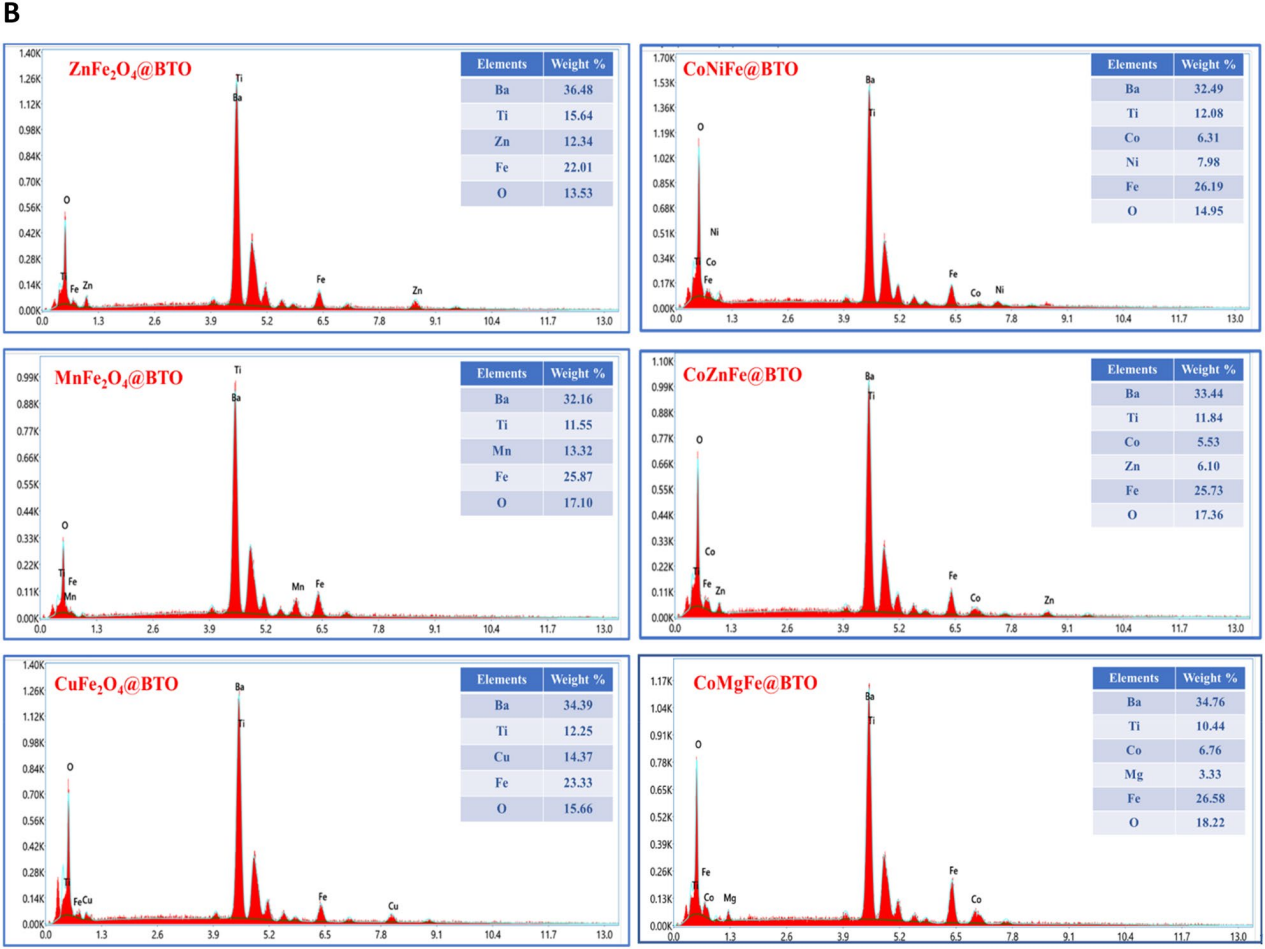
(**A**) SEM images of core–shell (**a**) ZnFe_2_O_4_@BTO, MnFe_2_O_4_@BTO and CoFe_2_O_4_@BTO (**b**) CoCuFe@BTO, CoMnFe@BTO and CoZnFe@BTO MENCs. (**B**) EDX spectra of core–shell (**a**) ZnFe_2_O_4_@BTO, MnFe_2_O_4_@BTO and CuFe_2_O_4_@BTO (**b**) CoNiFe@BTO, CoZnFe@BTO, and CoMgFe@BTO MENCs.

**Figure 3:**
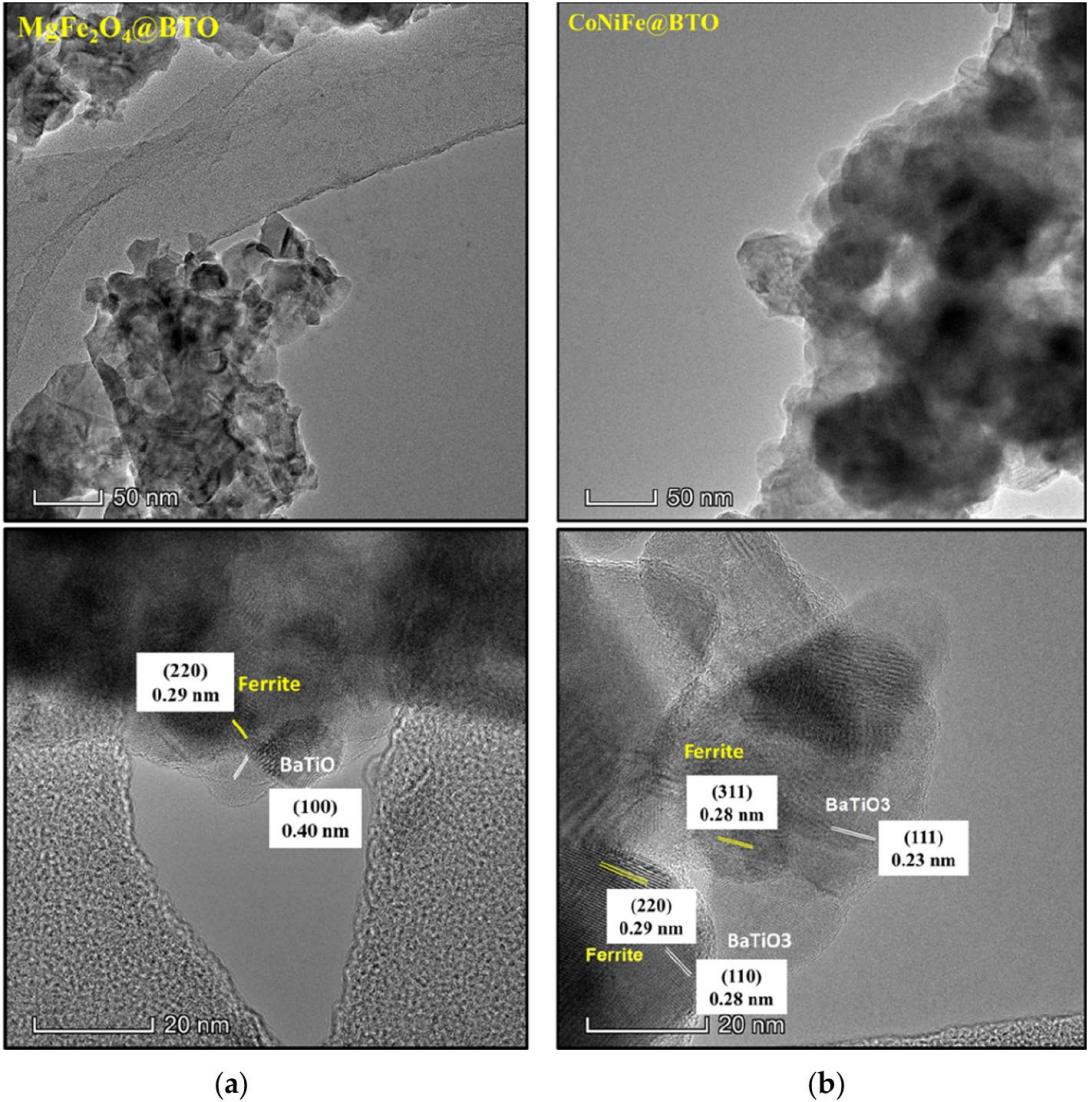
TEM and HR-TEM images of core–shell of (**a**) MgFe_2_O_4_@BTO, (**b**) CoNiFe@BTO MENCs.

The morphologies and microstructures of core–shell (ZnFe_2_O_4_@BTO, MnFe_2_O_4_@BTO, CoFe_2_O_4_@BTO, CoCuFe@BTO, CoMnFe@BTO, CoZnFe@BTO) MENCs were studied by SEM, and TEM. The SEM micrographs confirmed the spherical morphology of core–shell MENCs as presented in Fig. 2A. Samples exhibit nonuniformly diphase orientation (bright and medium dark regions) of agglomerated spherical grains. It is difficult to completely disperse the core material despite the vigorous ultrasonication dispersion of MNPs in BTO precursor solution during the coating process. Thus, they packed closely together due to their magnetic nature. Moreover, it is obvious the differences in the morphology with changing the core type due to the shape, the degree of agglomeration, and the different behavior of spinel ferrite MNPs in each composition. The elemental composition of core–shell (ZnFe_2_O_4_@BTO, MnFe_2_O_4_@BTO, CuFe_2_O_4_@BTO, CoNiFe@BTO, CoZnFe@BTO, and CoMgFe@BTO) MENCs was examined by EDX attached with SEM. The analysis was conducted to check the chemical purity of core–shell MENCs and their stoichiometry. The representative elemental compositions are shown in Fig. 2B. The EDX spectra emphasized the existence of the elements without any trace of impurities indicating the purity of the prepared samples. The TEM images stressed the formation of the core dark region (spinel ferrite MNPs phase) and the surrounding bright shell (BTO phase) as shown in Fig. 3. It can clearly distinguish the interface between two phases in the TEM images. The variation of core–shell color is due to the difference in transmission intensity and electron penetration efficiency on MNPs and BTO^43^. Moreover, the MNPs form agglomerates in BTO matrix. The corresponding high-resolution transmission electron microscopic (HR-TEM) images illustrate the well-defined lattice fringes of the magnetic core and BTO shell. The moire patterns are dominant in HR-TEM images which clearly exhibit the interference of crystallographic orientations of the ferrite and BTO phases. The crystallography of the two phases was proved by calculating the interplanar d-spacings that are in good agreement with planes of the ferrite phase and planes of the BTO phase. The interface between spinel ferrite and BTO phases is clearly shown by HR-TEM. Therefore, at this interface, the movement of strain between the ferrite and ferroelectric phase could happen and it might be suitable to build a strong ME coupling in the core–shell nanocomposite.

### Zeta potential measurements

The zeta potential is a valuable technique for assessing surface charge on the nanoparticles, predicting their stability and inferring the state of the surface^44^. Usually, nanoparticles having zeta potential in the range − 10 to + 10 has a neutral charge, while a zeta value higher than + 30 mV or lower than − 30 mV indicated a highly anionic and cationic surface respectively^38^. The zeta potential of MNPs and MENCs was studied and summarized in Table 2. It is clear from the zeta potential results that MnFe_2_O_4_ has the highest zeta potential as compared to other MNPs and MENCs, followed by CoFe_2_O_4_. CoMnFe showed the lowest zeta potential. Furthermore, results indicated that MNPs and MENCs have cationic surfaces^45^.

**Table 2:** Zeta potential of MNPs and MENCs.

Sample	Zeta potential (mV)
CoFe_2_O_4_	− 24.2
MnFe_2_O_4_	− 30.2
NiFe_2_O_4_@BTO	− 17.3
MnFe_2_O_4_@BTO	− 16.1
CoNiFe	22.1
CoMnFe	− 0.551

### Cytotoxicity in vitro examinations of MNPs and MENCs

#### MTT assay

**Figure 4:**
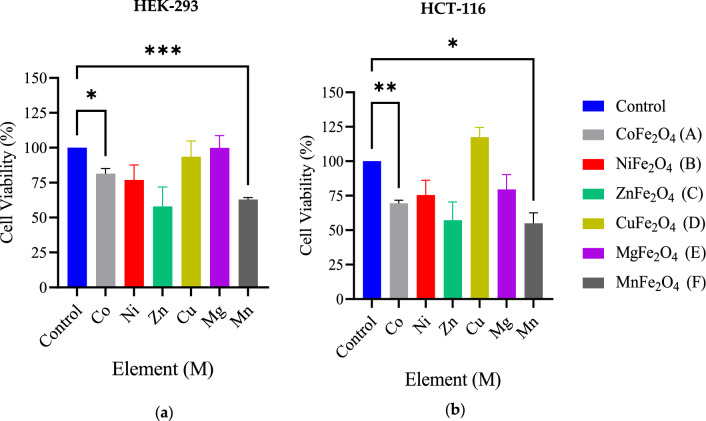
The average cell viability of (**a**) HEK-293 and (**b**) HCT-116 cell lines by MTT assay. Cells were treated with the following core composites MFe_2_O_4_ (M = Co, Ni, Mn, Mg, Zn, and Cu) MNPs and treatment concentration was 141.75 µg/ 0.1 ml for 48 h. n = 4 and error bars ± S.E.M. *p < 0.05; **p < 0.01; ***p < 0.001; versus control.

**Figure 5:**
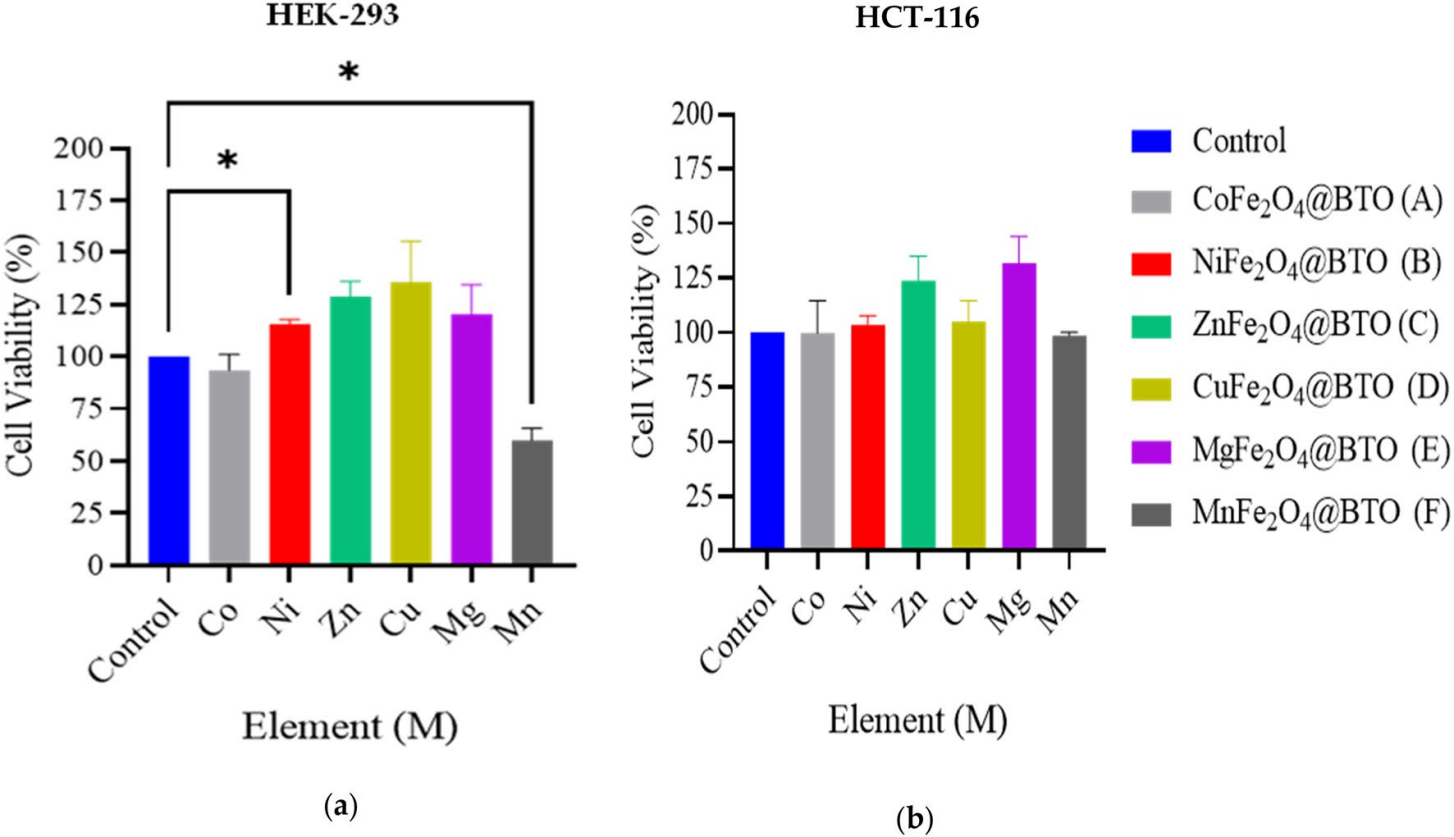
The average cell viability of (**a**) HEK-293 and (**b**) HCT-116 cell lines by MTT assay. Cells were treated with the following core composites MFe_2_ O_4_@BTO (M = Co, Ni, Mn, Mg, Zn, and Cu) and treatment concentration was 141.75 µg/ 0.1 ml for 48 h. n = 4 and error bars ± S.E.M. *p < 0.05; versus control.

In vitro analysis is an ideal model for human diseases study. It possesses high degree of transparency and the ability to identify a proper drug concentration for in vivo study as well as test the toxicity of the treated biomaterials on the cells. MTT assay is a common analysis technique conducted to examine the cytotoxicity of materials that demonstrate the dose–response relationship of the tested samples according to ISO standard 10993-5^46^. Therefore, we have examined the effect of spinel ferrite magnetic nanoparticles MNPs and core–shell MENCs on two different cell lines HCT-116 and HEK-293 through measuring mitochondrial reductase activity using 3[4,5-dimethylthiazol-2-yl]-2,5-diphenyl-tetrazolium bromide (MTT) as the substrate. Viable cells possess the ability to reduce MTT from a yellow water-soluble dye to an insoluble purple crystallized formazan product. Dimethyl Sulfoxide (DMSO) was used to dissolve the formazan crystals and quantified by measuring the light absorbance of the solution under the wavelength of 570 nm. The resultant value is correlated to the number of living cells. Figure 4 illustrates the significant reduction in normal cells HEK-293 and cancerous cells HCT-116 when treated with simple spinel ferrite MFe_2_O_4_ (M = Co, and Mn) at concentration of 141.75 µg/0.1 ml for 48 h. These magnetic cores revealed a toxic effect for both cell lines, and this could be explained by the presence of Co and Mn elements. The cellular system deals with iron and its oxide NPs as a part of iron physiology. Presumably, MNPs are degraded into iron ions under the influence of various hydrolyzing enzymes in the phagolysosomes at low pH as well as the proteins participating in iron metabolism and utilizing according to natural iron metabolism pathways^47,48^. Nevertheless, the degradation of CoFe_2_O_4_ within lysosome leads to slow etching and releasing of cobalt ions Co^2+^ where it is known to be toxic in larger doses^49,50^. Moreover, the cytotoxicity could be attributed to the ionization of metallic NPs inside the cells known as “Trojan-horse” mechanism according to Hsiao et al*.*^51^. Earlier studies have also shown toxicity of MNPs, Balakrishnan et al. revealed that CoFe_2_O_4_ exhibited moderate toxicity at 24 h, then it was gradually increased for 72 h incubation timeline^49^. M. Ahamed et al. have proved that CoFe_2_O_4_ NPs induced the cytotoxicity in the dose range 50–400 µg/ml in human liver cell line (HepG2) due to the production of reactive oxygen species (ROS)^52^. Another report confirmed that MnFe_2_O_4_ produced a various cellular damages and alterations causing cell death after entering breast cancer 4T1 cell^53^. CoFe_2_O_4_ coated with biocompatible BTO exhibited a protective effect for both cell lines. However, BTO was not able to protect the toxic effect of MnFe_2_O_4_ MNPs_,_ and MnFe_2_O_4_@BTO MENCs, it has shown a significant reduction after coating with BTO on HEK-293. The anti-apoptotic effect was observed with NiFe_2_O_4_@BTO MENCs on HEK-293 where it exhibited a significant increase in cell viability Fig. 5. Furthermore, the observed proliferation of HEK-293 is not significant, but it is suspicious when treated with MFe_2_O_4_@BTO (M = Zn, Cu, Mg) MENCs. Therefore, further experiments are required with different incubation time and different concentrations to understand the time and dose effecting manner.

**Figure 6:**
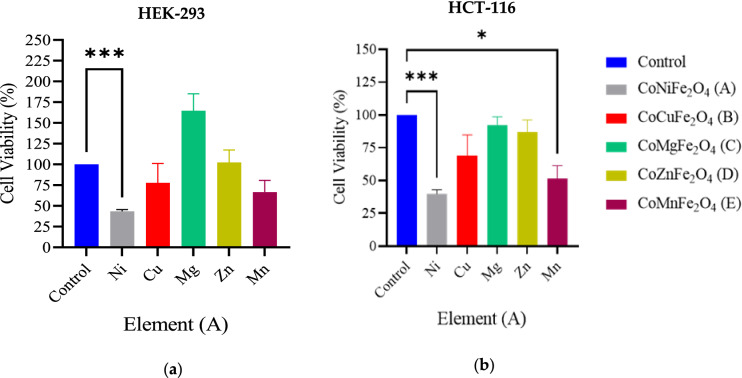
The average cell viability of (**a**) HEK-293 and (**b**) HCT-116 cell lines by MTT assay. Cells were treated with the following conditions CoMFe_2_O_4_ (M = Ni, Mn, Mg, Zn, and Cu) MNPs for 48 h. n = 4 dependent experiments. Error bars ± S.E.M. *p < 0.05; ***p < 0.001; versus control.

**Figure 7:**
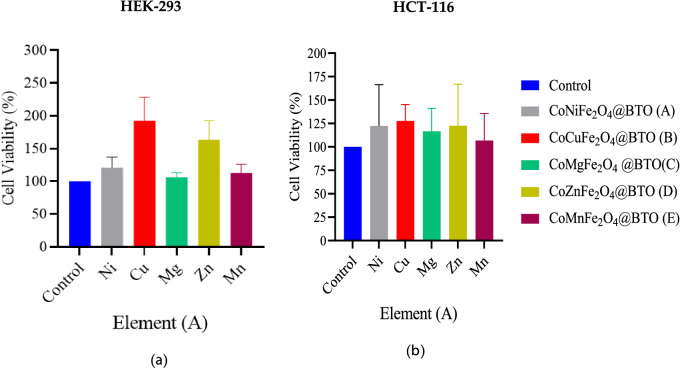
The average cell viability of (**a**) HEK-293 and (**b**) HCT-116 cell lines by MTT assay. Cells were treated with the following conditions CoMFe_2_O_4_@BTO (M = Ni, Mn, Mg, Zn, and Cu) MENCs for 48 h. n = 4 dependent experiments. Error bars ± S.E.M.

From the previous group we have selected CoFe_2_O_4_ MNPs and reduced the Co^2+^ concentration through doping with different transition metals CoMFe (M = Ni, Cu, Mg, Zn, and Mn) namely hard magnetic mixed ferrite for the toxicity reduction and physical properties enhancing. Both cell lines HCT-116 and HEK-293 were treated with CoMFe (M = Ni, Cu, Mg, Zn, and Mn) MNPs and CoMFe@BTO (M = Ni, Cu, Mg, Zn, and Mn) MENCs at concentration of 141.75 µg/0.1 ml. Post 48 h of treatment, the results revealed that CoNiFe exhibited a significant toxic effect for both cell lines in comparison with their control while CoMnFe showed selective statistically significant inhibitory effect *p* < 0.05 on colon cancer cells at the concentration of (141.75 µg/0.1 ml) as depicted in Fig. 6. These results suggested that CoMnFe can be a promising candidate for colon cancer treatment at 141.75 µg/0.1 ml concentration due to the induced selective toxicity on HCT-116 compared to control in vitro*.* The previous reports revealed that Ni NPs caused cytotoxicity in cancerous human lung epithelial A549 cells^54^. According to Freitas et al., the induction of the oxidative stress is the most frequently discussed mechanism for the Ni harmful effects through generation of ROS^55^. Herein, we expected the toxicity of CoNiFe MNPs due to the synergic effect of both meatal ions Co^2+^ and Ni^2+^. CoMgFe exhibited nonsignificant growth (*p* > 0.05) in normal cells HEK-293 and further experiments are necessary to confirm the result. Figure 7 shows the cell viability for both cell lines treated with CoMFe@BTO (M = Ni, Cu, Mg, Zn, and Mn) MENCs. The presence of BTO coating layer inhibited the toxic and pro-apoptotic effects of CoMFe. The results revealed that cell viability was more favorable in case of BTO coating CoMFe@BTO (M = Ni, Cu, Mg, Zn, and Mn) MENCs as shown in Fig. 7 than with uncoated ones. BTO exhibited recovery effect on HEK-293 and HCT-116 cells and no indication were observed of mass death of both cell lines which confirmed that CoMFe@BTO MENCs may not be toxic. Generally, we have observed that MENCs either maintain the cell viability or promote the cell proliferation within the certain composites. This may be related to the presence of BTO shell. It is a piezoelectric nanomaterial and possesses an ability to act as an active substrate to promote cellular growth under physiological environment^9^. BTO can generates an electric stimulation as response to transient structure deformation due to the migration and attachment of cells^8^. The generated electrical pulses are transmitted to the surrounding cells which promotes the cell signaling pathways and stimulates Ca^2+^-calmodulin pathway that responsible for synthesis the growth factor and enhance the cell growth^56,57^. G. Genchi et al. used BTO NPs to promote tissue regeneration. They have shown that the presence of BTO NPs in the scaffold was able to enhance the growth rate and proliferation of H9c2 myoblasts after 72 h^58^. BTO is the most promising nanomaterial with huge potential in a wide range of nanomedicine applications. Owing to its good biocompatibility, protectivity and its applicability in multifunctional theranostic systems including drug delivery, cell stimulation, and tissue engineering^58^.

#### The Impact of MNPs and MENCs on nuclear morphology

**Figure 8:**
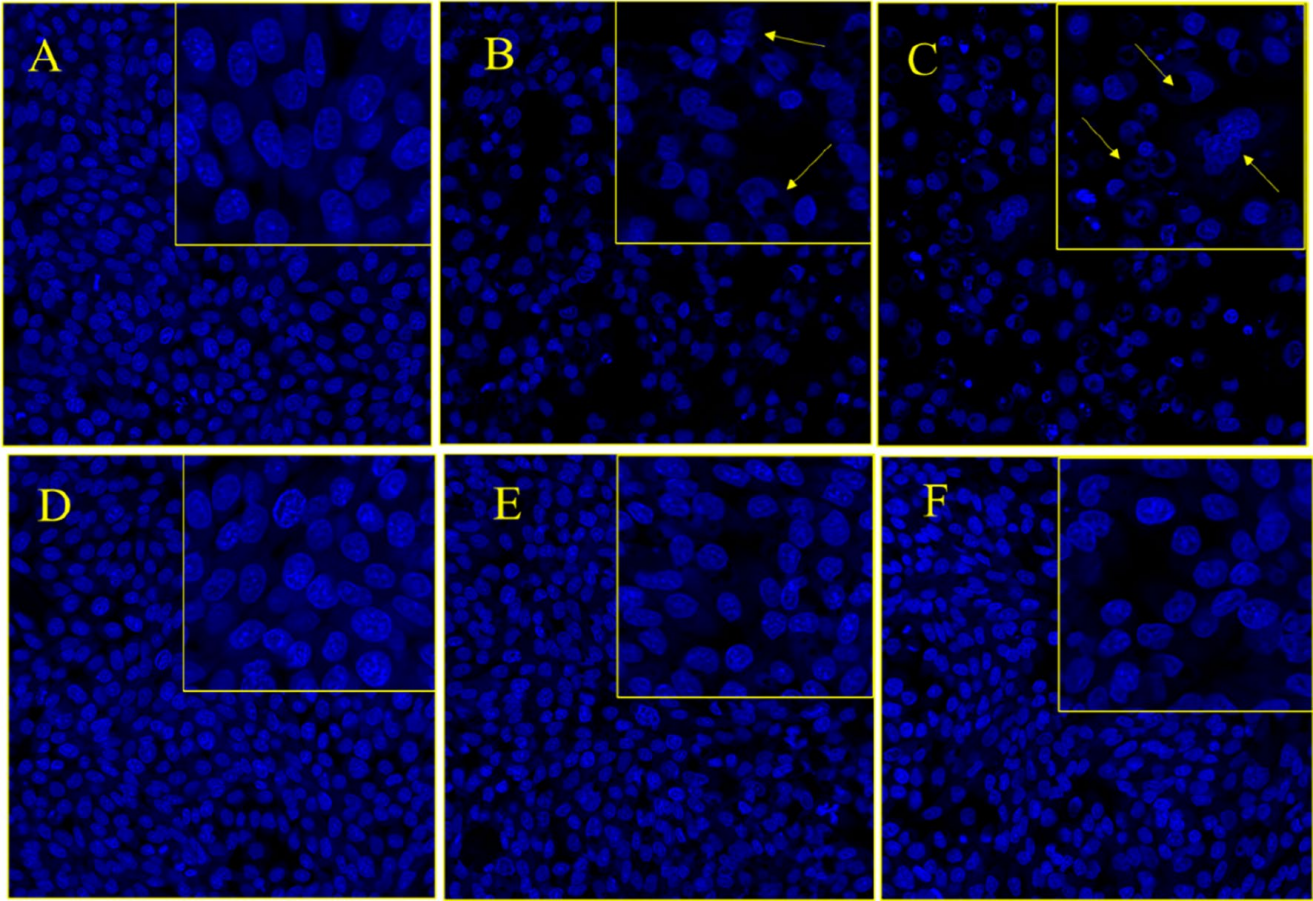
The impact of MNPs and MENCs treatment HCT-116 cells stained with DAPI after 48 h treatment. (**A**,**D**) are the control cell, (**B**) (CoFe_2_O_4_), (**C**) (MnFe_2_O_4_), (**E**) (CoFe2O4@BTO), and (**F**) (MnFe2O4@BTO). Arrows show the apoptosis signs.

The quantitative study was further augmented via the qualitative analysis of the cell nuclear morphology visualization under confocal microscope using DAPI (4′,6-diamidino-2-phenylindole) staining. It is fluorescent stain that binds very strongly to DNA and appears to associate with A-T rich regions in minor grove^59^. The passing of DAPI through live cell is less efficiently and therefore the effectiveness of the stain is low, thus cell must be permeabilize or fixed for the DAPI to enter the cell and bind with DNA. DAPI is normally used for cell counting, measuring apoptosis, and nuclear segmentation tool in high conducting imaging analysis. In this report, the colorectal carcinoma HCT-116 cells were stained with DAPI to visualize the impact of MNPs and MENCs on nuclear DNA. Also, it was used to identify the number of nuclei, visualization the apoptosis characteristic features include chromatin condensation, nuclear shrinkage and fragmentation, and to assess the gross cell morphology^31,60^. Figure 8B and C illustrates the inhibitory action on colon cancer cells due to treatment with CoFe_2_O_4_ and MnFe_2_O_4_ MNPs compared to control cells Fig. 8A. We have observed the apoptosis signs are dominant among the cells with clear reduction in cell number. On the other hand, we have observed minor cell death for MFe_2_O_4_@BTO (M = Co, Mn) due to the presence of biocompatible layer of BTO where it exhibited a recovery effect on cells (Fig. 8E,F).

**Figure 9:**
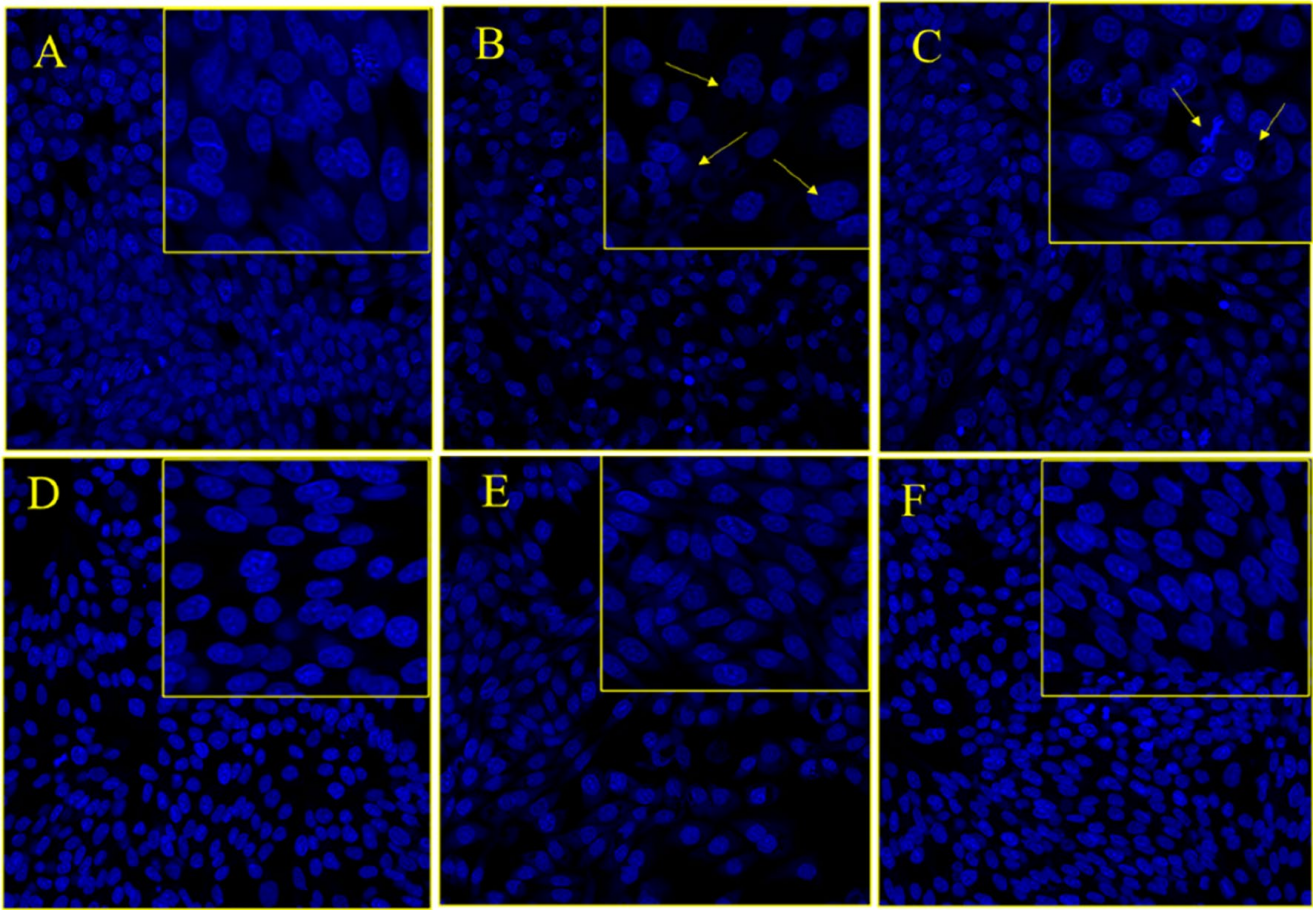
The impact of MNPs and MENCs treatment on HCT-116 cells stained with DAPI after 48-h treatment. (**A**,**D**) are the control cell, (**B**) (CoNiFe), (**C**) (CoMnFe), (**E**) (CoNiFe@BTO), and (**F**) (CoMnFe@BTO). Arrows show the apoptosis signs.

Similarly, HCT-116 cell lines were treated with CoMFe (M = Ni, Mn) MNPs and CoMFe@BTO (M = Ni, Mn) MENCs incubated for 48 h. In consistence with MTT results, we have observed that CoMnFe MNPs exhibited an inhibition action on cancer cells Fig. 9C; however, the effect was not strong as what we have seen with each individual composite MFe2O4 (M = Co, Mn). Moreover, there is a clear increase in cell deaths, nuclear condensation, and fragmentation in the CoNiFe MNPs treated cancer cells as shown in Fig. 9B. These findings suggest that CoMFe (M = Ni, Mn) MNPs promote cell death through the proapoptotic effect. The BTO coating layer have relieved the inhibition effect of MNPs. Figure 9E and F revealed that the nuclei possess a close morphology to control Fig. 9D with minimum cell reduction as well as the apoptosis signs. The control cells remained intact and neither show any nuclear condensation, nor cell membrane disintegration and cell death as shown in Figs. 8A,D and 9A,D.

#### Erythrocyte lysis assay

**Figure 10:**
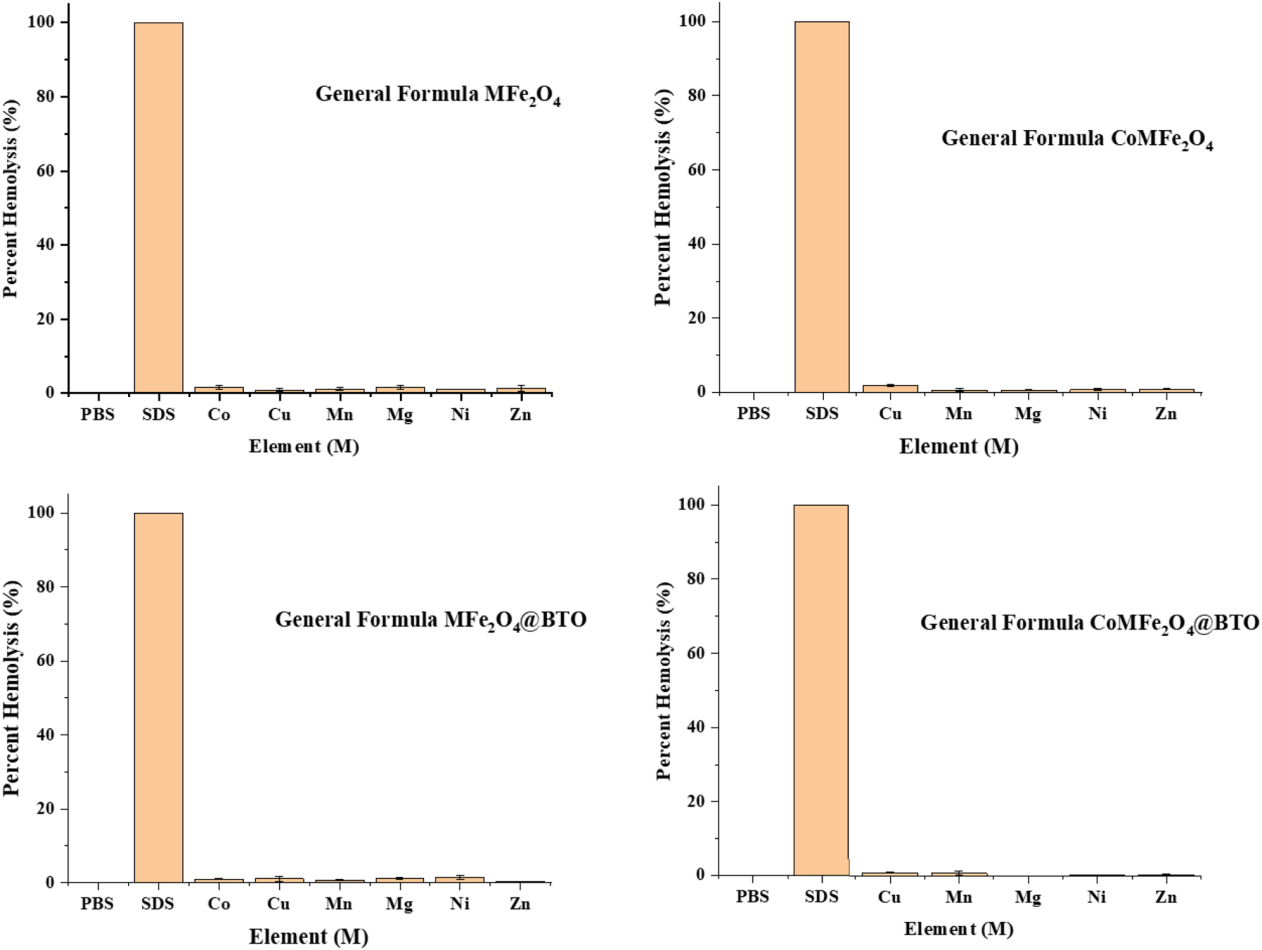
Shows hemolytic effect of (MFe_2_O_4_, CoMFe_2_O_4_; M = Ni, Co, Mn, Mg, Zn, and Cu) MNPs and (MFe_2_O_4_@BTO, CoMFe_2_O_4_@BTO; M = Ni, Co, Mn, Mg, Zn, and Cu) MENCs at the lowest concentration of 33 µg/0.1 ml. Data represent mean ± S.E.M. of two individual experiments. Normal blood sample in PBS used as negative control. While SDS is the hemolysis of positive control which was more than 80%.

**Figure 11:**
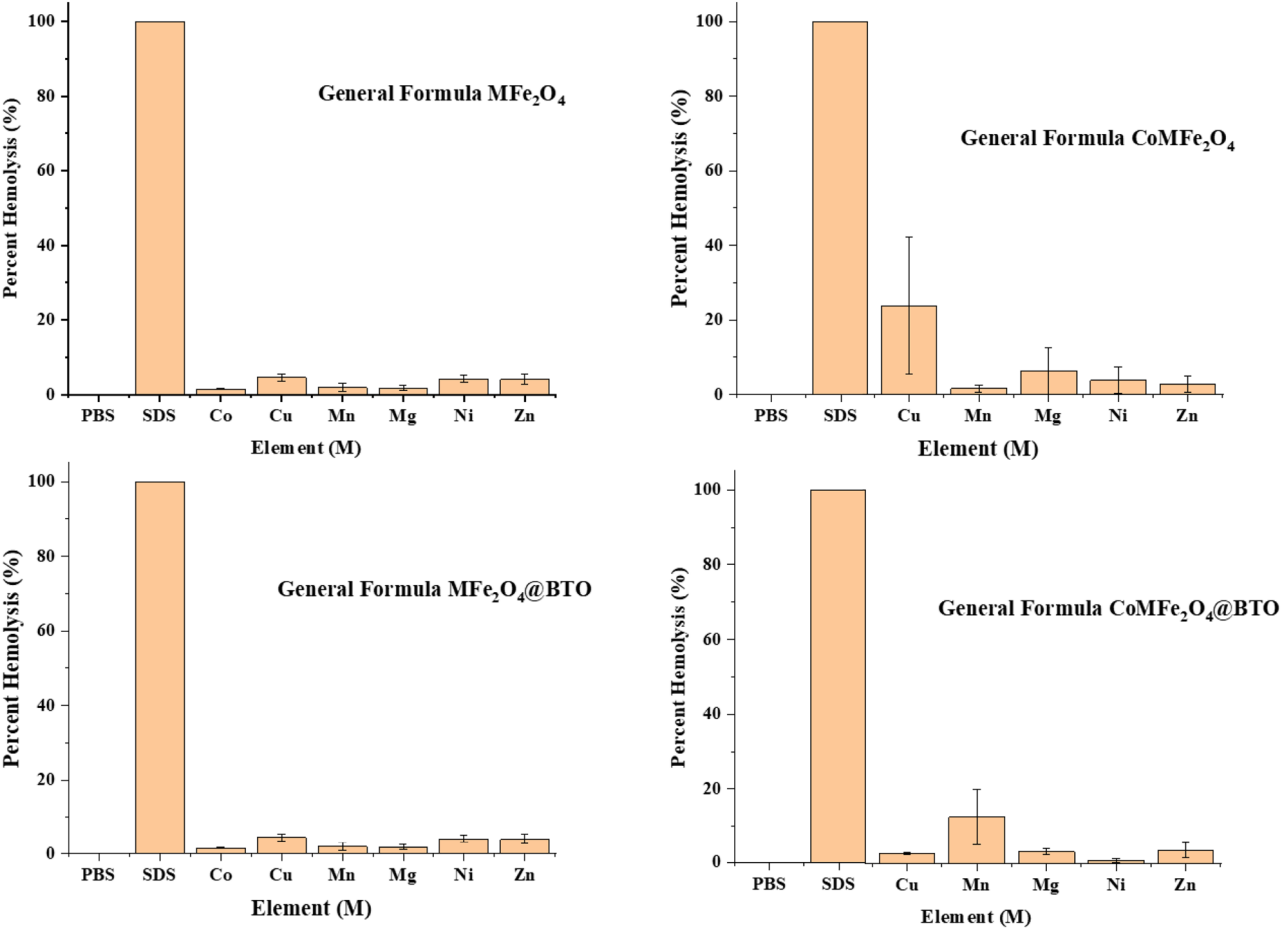
Shows hemolytic effect of (MFe_2_O_4_, CoMFe_2_O_4_; M = Ni, Co, Mn, Mg, Zn, and Cu) MNPs and (MFe_2_O_4_@BTO, CoMFe_2_O_4_@BTO; M = Ni, Co, Mn, Mg, Zn, and Cu) MENCs at the highest concentration of 276 µg/0.1 ml. Data represent mean ± S.E.M. of two individual experiments. Normal blood sample in PBS used as negative control. While SDS is the hemolysis of positive control which was more than 80%.

**Figure 12:**
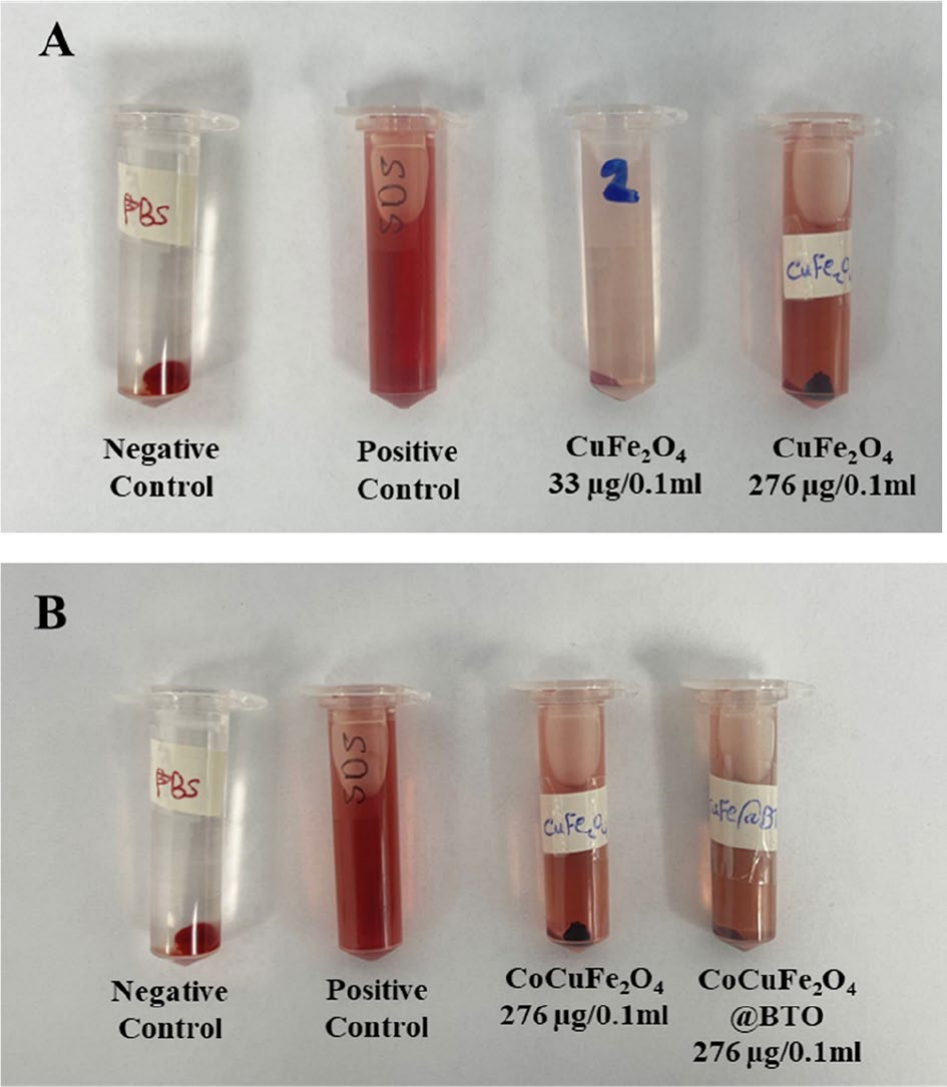
Shows the visualization of hemolytic effect. (**A**) Comparison between the lowest and highest concentration of (CuFe_2_O_4_) with respect to positive and negative control. (**B**) The effect of the presence BTO biocompatible layer in CoCuFe_2_O_4_ at the highest concentration 276 µg/0.1 ml.

The hemolytic potential assay has been conducted to assess the toxicity of different MNPs and MENCs formulations at the cellular level as illustrated in Figs. 10 and 11. According to ISO 10993-4 which stands for the blood compatibility evaluation of the medical devices contain or generate nanomaterials. The standard states the following criteria of hemolysis percentage where (0–2%) is nonhemolytic biomaterial, (2–5%) slightly hemolytic, or (> 5%) hemolytic^29^. It has been observed that all the formulation in this study at the lowest concentration 33 µg/0.1 ml either core (MFe_2_O_4_, CoMFe_2_O_4_; M = Ni, Co, Mn, Mg, Zn, and Cu) MNPs or core–shell (MFe_2_O_4_@BTO, CoMFe_2_O_4_@BTO; M = Ni, Co, Mn, Mg, Zn, and Cu) MENCs showed nonhemolytic effect (0–2%). In contrast, the highest concentration 276 µg/0.1 ml exhibited a slightly to high hemolytic effect (> 5%) as detailed in Table 3 and Fig. 12. Upon close analysis, the presence of a biocompatible BTO layer plays a crucial role in terms of reducing the hemolytic effect of different core formulations even with the highest concentration as shown in Fig. 12. The large surface-to-volume ratio is one of the most important parameters of NPs where the smaller size of particle, the larger surface area they have. Although NPs possess the advantage of large loading drug due to large surface area, however; they promote the reaction of oxygen with tissues and creating free radicals^47^ which is oxidative stress factor on the cell. It has been acknowledged from literatures that the cytotoxicity and human cells apoptosis are generally based on the ROS production and oxidative stress due to the exposing to MNPs^61–63^. Several studies reported that the blocking of nanoparticles ROS leads to minimize their interaction with RBCs membrane and therefore their potential hemolytic effect^64^. Therefore, uncoated MNPs might be cytotoxic due to the direct contact with cells^65^.

**Table 3:** Summary of hemolysis and cytotoxicity effects of MNPs and MENCs on RBCs, human embryonic kidney HEK-293 and human colorectal cancer HCT-116 cell lines.

Conc	Sample group	Hemolysis classification			MTT assay	HEK-293			HCT-116		
		Nonhemolytic (0–2%)	Slightly hemolytic (2–5%)	Hemolytic (> 5%)		*	**	***	*	**	***
Low 33 µg/0.1 ml	MFe_2_O_4_	All			Inhibition	Co		Mn	Mn	Co	
	CoMFe_2_O_4_	All						Ni	Mn		Ni
	MFe_2_O_4_@BTO	All				Mn					
	CoMFe_2_O_4_@BTO	All									
High 276 µg/0.1 ml	MFe_2_O_4_	Mn	Co, Cu	Mg, Ni, Zn	Proliferation						
	CoMFe_2_O_4_	Mn	Ni, Zn	Cu, Mg							
	MFe_2_O_4_@BTO	Co, Mn, Mg	Cu, Ni, Zn			Ni					
	CoMFe_2_O_4_@BTO	Ni	Cu, Mg, Zn	Mn

#### Comparison in the biological activities of MNPs and MENCs

The NPs cytotoxicity and adverse hematology effect depend on various particle parameters. The main influencing factors are materials’ morphology, size, composition, hydrophobicity, surface area, and surface charge^29^. On the other hand, different biological parameters influence cytotoxicity like cell type, culture and exposure conditions (i.e. cell density, particle concentration, and temperature^66^. In addition to oxidative stress, the other mechanisms of toxicity and forms of injuries might be resulted from NPs interaction include protein denaturation, membrane damage, DNA damage, and immune reactivity^67^. Or analysis of lysosomes membrane which lead to leaking of analytical enzymes into the cell resulting in cell apoptosis^68^. The obtained hemolysis and cytotoxicity results are summarized in Table 3. Commonly, inverse structure magnetic ferrite exhibited an obvious reduction in cell viability, while normal structure magnetic ferrite showed an opposite action through maintaining the cell viability or promoting the cellular growth. These findings can be explained by the spinel ferrite MNPs activity where it depends on different parameters such as particle size, surface texturing, stability, metal ions redox properties, and cations distribution among tetrahedral and octahedral sites^69^. CoFe_2_O_4_ belongs to inverse spinel ferrites were Fe^3+^ have tetrahedral coordination and (Co^2+^) and (Fe^3+^) are equally distributed in octahedral sites^70^. The spinal’s ferrite MNPs surfaces mainly composed of octahedral sites. According to the previous reports, the metal ions that occupied the octahedral positions play a crucial role in the catalytic activity due to the longer bond length; thus, it can be easily interact with the reactant molecules^69,71,72^. However, the metal ions that occupied the tetrahedral sites are rarely contributed to the reduction activity. The inactivity of this crystallite coordinate site can be originated from the strong metal–oxygen bonds because of the lower valency and coordination number. Furthermore, the tetrahedral cations are not freely accessible to the reactants^73^. Ibrahim et al*.* have reported that the catalytic reaction was the highest in case of MnFe_2_O_4_ compared to CoFe_2_O_4_ both exceeded that of ZnFe_2_O_4_. They argued that this is due to the presence of (Mn^2+^ and Fe^3+^) or (Co^2+^ and Fe^3+^) ions in the octahedral positions of the ferrite sublattice while in ZnFe_2_O_4_ the only Fe^3+^ ions are present^69^. Upon close investigations, we have found that the simple ferrite MNPs CoFe_2_O_4_ and MnFe_2_O_4_ exhibited a toxic effect on both cell line; however, the composite of CoMnFe has shown a remarkable selective anticancer effect on HCT-116 as depicted in Table 3. Moreover, CoMnFe MNPs has shown the nonhemolytic effect even at highest concentration 276 µg/0.1 ml, while the CoFe_2_O_4_ MNPs at the same concentration exhibited slightly hemolytic effect. This can be attributed to the different catalytic action of simple and mixed magnetic ferrite which is correlated to the electronic structure as well as the synergic interaction between different metals^74^. Moreover, this could be correlated to the surface charge from zeta potential measurements in Table 2 where the CoMnFe possesses the stable and lowest zeta potential in comparison with CoFe_2_O_4_ and MnFe_2_O_4_.

## Materials and methods

### Preparation of MNPs

MNPs were prepared using the ultrasonic irradiation technique. These reagents (Ni(NO_3_)_2_·6H_2_O) nickel nitrate, (Zn(NO_3_)_2_·6H_2_O) zinc nitrate hexahydrate, (Cu(NO_3_)_2_·H_2_O) copper nitrate tetrahydrate, (Fe(NO_3_)_2_·9H_2_O) iron nitrate nonahydrate, (Co(NO_3_)_2_·6H_2_O) cobalt nitrate hexahydrate, (Mn(NO_3_)_2_·6H_2_O) manganese nitrate hexahydrate, (Mg(NO_3_)_2_·6H_2_O) magnesium nitrate hexahydrate, (Ca(NO_3_)_2_·4H_2_O) calcium nitrate tetrahydrate, were employed as the starting preparation materials. A proper stoichiometric from each material has been taken and mixed in deionized water under continuous stirring to prepare the separate spinel ferrites.

Once we got a homogenous metal solution, the pH was arranged equally to 11 by using 2 M NaOH solution. The sonication probe (Ultrasonic homogenizer UZ SONOPULS HD 2070 with a power of 70 W and a frequency of 20 kHz) was used to conduct the reaction for 1 h. The obtained product was washed several times with hot deionized water. Then it was dried at 180 °C for 12 h and crushed in an agate mortar to get MNPs.

### Preparation of core–shell MENCs

**Figure 13:**
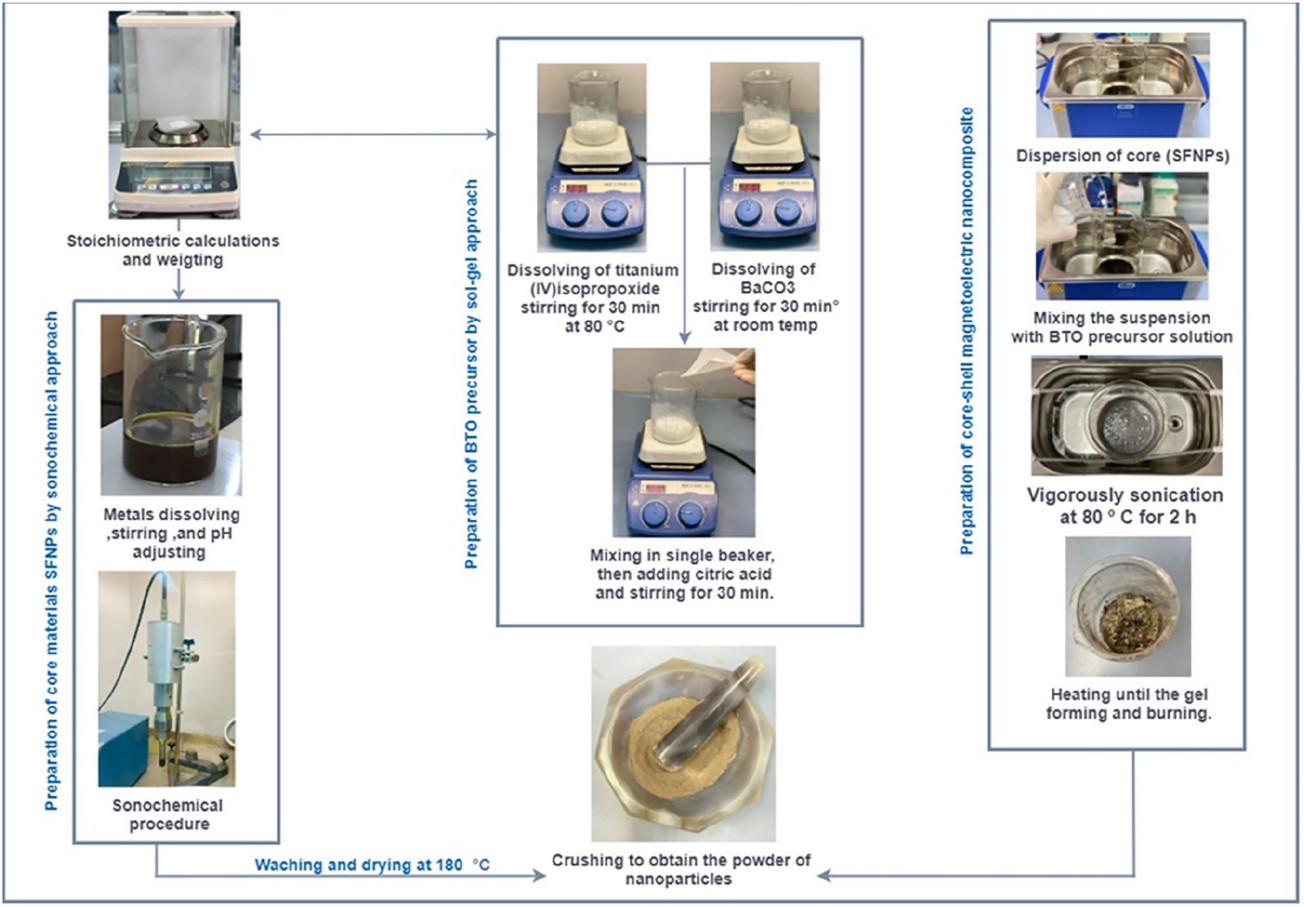
The experimental procedure scheme for MNPs and MENCs preparation process.

The citrate sol–gel auto-combustion procedure was used to prepare MENCs. Firstly, 1.9 g of barium carbonate was mixed with 10 ml of deionized water and 10 ml of ethanol with continuous stirring for 20 min. Similarly, 2.8 ml of titanium (IV) isopropoxide was mixed with 50 ml ethanol and 50 ml deionized water with continuous heating and stirring at 80 °C temperature and 30 min, respectively. In a separate beaker, these two prepared solutions were mixed then 4.2 g citric acid was added and placed on a hot plate at (80 °C) with stirring for 20 min. The as-prepared MNPs were dispersed in 20 ml of ethanol using a sonication bath for 30 min at room temperature. Later, the MNPs suspension was mixed with prepared BTO precursor solution and then placed in the sonication bath for vigorous vibration at 80 °C for 2 h. Finally, the resultant product was retained on the hot plate at 80 °C and kept until the solution becomes thick white near to gel. Then, the temperature was raised to 120 °C to burn the formed gel. Subsequently, the received powder was grounded and then calcined in a muffle furnace at 800 °C for 5 h to obtain core–shell MENCs powder. Figure 13 illustrates the schematic sequence of the experimental procedure.

### Characterizations of core–shell MENCs

The crystal microstructure was performed by a Rigaku Benchtop Miniflex X-ray diffraction (XRD, Cu Kα radiation) at room temperature. Rietveld refinement was conducted to determine the phases of the prepared samples through comparing the experimental diffraction patterns with the standard database via phase analysis software (Match3! and Fullproof). The imaging techniques include Scanning Electron Microscopy (SEM) combined with Energy Dispersive X-ray Spectroscopy (EDX) system, and Transmission Electron Microscope (TEM) were employed to examine the composites' surface morphology. Zeta Potential of the MNPs and MENCs in DI water was measured by dynamic light scattering (DLS) (ZEN5600, Malvern, UK).

### In vitro cytotoxicity examination

In this study, we have used normal healthy human embryonic kidney cells (HEK-293) and human colorectal carcinoma cells (HCT-116) which were purchased from ATCC (American Type Culture Collection), Manassas, Virginia, USA to assess the influence of MNPs and MENCs. The colorimetric MTT assay was utilized to measure the cell viability as explained previously^75^. In brief, cells which have more than 80% confluence were trypsinized and counted. Thereafter, cells were seeded in 96-well plates then treated with different concentrations (33–267 µg/0.1 ml) of MNPs and MENCs, except the control group. Post 48 h, the cells were treated with MTT (5 mg/ml) solution and preserved for 4 h. Lastly, the cells were washed and examined at 570 nm wavelength through microplate reader (Biotek Instruments, Winooski, USA).

### DAPI staining

Colorectal carcinoma cells (HCT-116) were stained with DAPI to visualize the impact of MNPs and MENCs on nuclear DNA of cancer cells. HCT-116 cells were seeded in chamber slides in CO_2_ incubator (5%) at temperature of 37 °C, allowed to attach overnight. Then, cells were separated into two groups: one was untreated control group and another one was treated with (88.8 µg/0.1 ml) dosage of MNPs and MENCs. Post 48 h, both groups were treated with ice-cold paraformaldehyde (4%) solution then washed with PBS. Thereafter, cells were labelled with DAPI under a dark environment and kept for 30 min. Lastly, the cells were washed in PBS and their morphology was visualized using laser Confocal Scanning Microscope (Zeiss, Frankfurt, Germany).

### Erythrocyte lysis assay

The erythrocyte lysis assay was conducted according to Shivashankarappa et al.^76^. The spectrophotometer was utilized to examine the cytotoxicity by the measuring the amount of hemoglobin released via RBC’s membrane rupture. The fresh blood was taken from adult wistar rat and EDTA was added to the collecting tube to prevent blood coagulation. It was centrifuged for 10 min at 1500 rpm at 4 °C and the plasma with white layer containing WBC and platelets was removed carefully by aspiration. Thereafter, the erythrocytes pellets were washed three times with PBS (pH 7.4) and resuspended in PBS to give nine times its volume. Two different concentrations (lowest 33 µg/0.1 ml and highest 267 µg/0.1 ml) of MNPs and MENCs were used for RBCs treatment and the PBS was added to reach the total volume of 2 ml. Then, it was incubated for 20 min at 37 °C followed by centrifuging at 2000 rpm for 3 min. The supernatant was collected, and the density of the color measured at 540 UV visible spectrophotometer. 1% SDS was used as a positive control, and the PBS was used as a negative control. The percent of hemolysis was calculated according to the following formula^77^:

$${\text{Percent of hemolysis }}({\%}) =\frac{Negative \; control-Test \; sample}{Positive \; control-Negative \;  control}$$

### Statistical analysis

All statistical analyses were run on GraphPad Prism Software [Version 9.0]. Mean ± standard error (S.E.M) from control, MNPs and MENCs was calculated. One-way analysis of variance (ANOVA) with Dunnett’s post hoc test were used to calculate the difference between control and NPs treated groups. Error bars ± S.E.M. *p < 0.05; **p < 0.01; ***p < 0.001 versus control.

### Consent for publication

All authors have read and agreed the final draft of the manuscript for consideration for publication.

## Conclusions

In the present study, we have used sonochemical and sol–gel techniques to prepare various (MFe_1.8_O_4_, CoMFe) MNPs and core–shell (MFe_1.8_O_4_@BTO, CoMFe@BTO,) MENCs. XRD analysis confirmed the purity of all products (MNPs and MENCs) and the average crystallite size of core–shell MENCs which was evaluated within 24–45 nm range. The morphology analyses (both TEM and SEM) revealed the aggregated spherical grains with different agglomeration degree with various spinel ferrite magnetic core. Core–shell MENCs were designed to overcome the disadvantages that associated with MNPs in term of physical and biological enhancement. It was proved that magnetic core coated with BTO matrix is biocompatible. Moreover, the usage of MENCs in cancer therapy do not require heat generation which could potentially damage the surrounding healthy tissue. They can efficiently release drug in controlled protocol independent of physiological changes in the presence of magnetic field. We have also evaluated the biological impact of (MFe_1.8_O_4_, CoMFe) MNPs and core–shell (MFe_1.8_O_4_@BTO, CoMFe@BTO) MECNs on normal HEK-293 and cancerous HCT-116 cell lines by MTT assay and DAPI staining. After 48 h of treatment, the results of the MTT assay have shown that hard magnetic mixed ferrite CoMFe (M = Ni, Cu, Mg, Mn, Zn) exhibited an anti-proliferative effect. It was very observable on colon cancer cells HCT-116 where the significant reduction was obvious in CoMnFe with spiring the normal cells. Each CoFe_2_O_4_ NCs and MnFe_2_O_4_ revealed a toxic effect for both cell lines while the CoMnFe NCs exhibited the selective anti-cancer action on colorectal cancer cells due to the metals’ synergic effect and the electronic structure differences. Consequently, the CoNiFe NCs possess a highly toxic effect for both cell lines thus it is not recommended in biomedical applications. The coating of MNPs with biocompatible BTO layer reduce the pro-apoptotic effect of magnetic core. MENCs eliminated the direct contact of uncoated MNPs with cells, therefore it relived the toxicity of MNPs. RBCs hemolytic effect of NPs has ranged from non- to low-hemolytic effect. This effect that could be attributed to the surface charge from zeta potential the CoMnFe possesses the stable and lowest zeta potential in comparison with CoFe_2_O_4_ and MnFe_2_O_4_. Also, to the protective effect of shell. Further examinations are required to investigate the cellular effect in different incubation time, concentrations, and to ensure the cytocompatibility and carcinogenicity of MNPs and MENCs. This study was conducted and applied on in vitro, so applying it in future in vivo studies is highly recommended. Developing a high quality magnetoelectric materials, with suitable structure, morphology, particles size, surface charges and minimum denaturation with the lowest cytotoxic effect is a demanding plan for anti-cancer drugs and drug carriers. So, using certain formulations with BTO is a promising strategy targeting cancer.

## Data Availability

All data generated or analyzed during this study are available within this manuscript.
